# General population normative data from seven European countries for the K10 and K6 scales for psychological distress

**DOI:** 10.1038/s41598-023-45124-0

**Published:** 2023-10-26

**Authors:** J. Lehmann, M. J. Pilz, B. Holzner, G. Kemmler, J. M. Giesinger

**Affiliations:** 1 Health Outcomes Research Unit, University Hospital of Psychiatry II, Medical University of Innsbruck, Innrain 43, 6020 Innsbruck, Austria; 2 University Hospital of Psychiatry I, Medical University of Innsbruck, Innsbruck, Austria

**Keywords:** Psychology, Medical research, Psychiatric disorders

## Abstract

The 10-item Kessler Psychological Distress scale (K10) and its 6-item short-form version (K6) measure psychological distress, particularly anxiety or depressive symptoms. While these questionnaire scales are widely used in various settings and populations, general population normative data are rarely available. To facilitate the interpretation of K10 and K6 scores, we provide normative general population data from seven European countries. We used an online survey to collect K10 data from general population samples in Austria, Italy, Germany, France, the Netherlands, Poland and Spain. We calculated the age- and sex-specific normative values separately for each country. For more specific estimates of K10 and K6 scores for individuals or groups, we also established a multivariable regression model based on socio-demographic and health data. In total, N = 7,087 adults participated in our study (51.6% women; mean age, 49.6 years). The mean K10 score in the total sample was 8.5 points (standard deviation, 7.3) on 0–40 points metric, with mean scores in individual countries ranging from 6.9 (the Netherlands) to 9.9 (Spain). Women showed higher scores than men and younger participants scored higher than older participants. Our study is the first to present normative K10 and K6 data from several European countries using a consistent sampling approach. These reference values will facilitate the interpretation of K10 and K6 scores in clinical research and practice and also highlight the variation in psychological distress levels across countries and groups according to their socio-demographic and health characteristics.

## Introduction

Affective and anxiety disorders are the most common psychiatric diagnoses in the general population^1,2^. The Organisation for Economic Co-operation and Development’s 2018 Health at a Glance report^3^ estimates that 25 million people (5.4% of the general population) in the European Union were living with an anxiety disorder and more than 21 million people (4.5% of the general population) were living with depressive disorders, which not only cause individual suffering but also high socio-economic costs^3^. While these prevalence rates are high, they may still underestimate the true extent of such mental health problems because these are known to be underdiagnosed and can exist for a long time before being detected and treated^4^. Effective diagnostic strategies are therefore of vital importance, not only for obtaining reliable prevalence estimates but also better referral of individuals to adequate treatment.

Structured diagnostic interviews for diagnosis of mental health disorders, such as the Structured Clinical Interview for DSM Disorders (SCID)^5^, or the Composite International Diagnostic Interview (CIDI)^6^ are considered the diagnostic gold standard but take a long time to complete and can only be conducted in a one-on-one setting. In contrast, screening questionnaires targeting mental health disorders offer the benefits of being concise and applicable in a wider research context without the need for direct contact with a mental health professional. Such questionnaires allow a detailed assessment of psychological distress levels and may also help to identify individuals with mental health disorders.

While numerous psychological screening questionnaires are available for specific patient populations and target disorders^7–9^, the 10-item Kessler Psychological Distress scale (K10)^10^ was developed specifically to assess psychological distress and screen for mental health disorders in the general population. Initially, its unidimensional scale intended to measure “psychological distress”^10^, whereby depression and anxiety were identified as secondary independent factors in multifactorial models^11^. Since its development, the K10 scale has been used in a number of large-scale epidemiological studies^12–14^ and in clinical practice^15,16^, in addition to measuring clinical study outcomes^17^. The K10 scale and its short-form version, the K6 scale, have shown strong psychometric properties such as good reliability, construct and criterion validity in various populations^18–20^, with some variation across different cultural groups^15,21,22^ Furthermore, they possess the ability to identify individuals with mental health disorders in different settings with high accuracy^12,23–25^. For screening purposes, thresholds have been established to allow the calculation of the prevalence rates of mental health disorders. While prevalence data are easy to interpret, they come at the disadvantage of reduced information because the actual distribution of the varying levels of distress is lost when thresholds are applied. The use of normative data, such as general population data, is an alternative approach for the interpretation of scale scores that does not require thresholds. The measurement of these normative data for psychological distress using the K10 scale provides detailed information for researchers, health-care professionals, and policymakers about the distress levels in different groups of individuals. These data may help to identify vulnerable populations. Previous studies have collected reference data from large samples of diverse populations^13,14,26^, but general population normative data are rarely available. Considering the variation in the score distributions and measurement characteristics of the K10 scale^15^ across countries, country-specific normative data should allow for the valid interpretation of scores.

Therefore, to facilitate the interpretation of K10 (and K6) data from European populations and to investigate the variation in K10 (and K6) scores across countries, our study aimed to establish sex- and age-specific general population normative data from seven European countries (i.e., Austria, France, Germany, Italy, the Netherlands, Poland, and Spain).

## Methods

### Sample

This study used adult general population data from a cross-cultural study in seven European countries^27–29^ to obtain normative values for the K10 scale. We outsourced the panel data collection to a market research institute, SurveyEngine (Berlin, Germany, https://surveyengine.com/), which contacts panel members who have registered voluntarily and agreed to participate in similar studies. The countries were assessed in consecutive projects; therefore, the online surveys were sent out between September 2015 and December 2018. We set quotas for sex and the predefined age groups (18–29, 30–39, 40–49, 50–59, 60–69, and 70 + years) to obtain a raw approximation of the proportion of the general population in these age and sex groups based on United Nations statistics^30^.

### Socio-demographic and health data

The questionnaire included a data form that collected the participants’ basic socio-demographic and health data, including their age, sex, educational level, marital status and living situation. We also asked the participants if they had been hospitalised during the previous 12 months and if they suffer from health conditions. For the latter, we provided a list of major chronic disorders, including mental health disorder, with a binary response format (i.e., no/yes) for each of these conditions.

### The K10 and K6 scales

The K10 comprises 10 items exploring the non-specific psychological distress experienced in the last 4 weeks^10^. In addition, the K6 scale uses the first six items from the K10 scale. Both questionnaire versions can be used to indicate distress in populations or individuals. All items are scored on a 5-point Likert scale (1 = ‘none of the time’ to 5 = ‘all of the time’). All items assess the participants’ psychological distress with questions focusing on anxiety and depression, such as, ‘In the last 4 weeks, how often did you feel nervous?’.

A total score can be calculated by adding all item scores, with high scores indicating high levels of distress. Following the original scoring instructions^10^, the score range for the K10 is 0 to 40 points, while the score range for the K6 short-form is 0–24 points.

### Statistical analysis

Sample characteristics are given as means, standard deviations, and absolute and relative frequencies. While the data collection already approximated the age and sex distribution in the individual countries, we applied additional weights using raking to more precisely match the national age and sex distributions^30^.

We described the weighted normative data for the K10 and K6 scales using means and standard deviations (SDs) and percentiles (10th, 25th, 50th, 75th and 90th) separately for the total sample and country-, age- and sex-specific groups.

To allow for more precise normative values in specific groups of individuals, we also developed a regression model to predict their K10 and K6 scores using the following independent variables: sex, age group, educational level, somatic chronic conditions, mental chronic conditions, and country. All predictors that were statistically significant in the univariate analysis (*p* < 0.05) were included in the multivariable model, except for mental health, which we excluded from the multivariate analysis to avoid over-adjustment.

To evaluate the diagnostic accuracy of the K10 scale in predicting self-reported mental health disorders (as reported in the initial questionnaire data), we used receiver operating characteristic (ROC) analysis to calculate the area under the curve (AUC) as a measure of diagnostic accuracy and determined the possible cut-off values separately for each country.

### Ethical approval and consent to participate

Data is not publicly available, but was provided anonymised by the panel research company SurveyEngine GmbH to the authors. No ethics approval was sought as the study is based on panel data. According to the NHS Health Research Authority and the European Pharmaceutical Market Research Association (EphMRA), panel research does not require ethical approval if ethical guidelines are followed. The survey was distributed via the SurveyEngine GmbH and obtained informed consent by each participant before the study. All data were collected anonymously and identification of the respondents through the authors or anyone else is impossible. The authors assert that all procedures contributing to this work comply with the ethical standards of the relevant national and institutional committees on human experimentation and with the Helsinki Declaration of 1975, as revised in 2008. The authors assert that all procedures contributing to this work comply with the ethical standards of the relevant national and institutional guides on the care and use of laboratory animals.

## Results

### Participant characteristics

The survey data from N = 7,087 adult individuals from seven European countries were available for analysis. In the unweighted data, the mean age was 49.6 (SD = 16.1) and women comprised 51.6% of the sample. The weights applied to the data ranged from 0.74 to 1.83 units. In the weighted data, the mean age was 50.0 years (SD = 16.6) and women still comprised 51.6% of the sample. Most participants indicated an educational level of secondary school or vocational training (56.3%). Health conditions were reported by 39.4% of the participants, with arthritis/rheumatism (11.0%), asthma/chronic obstructive pulmonary disease (9.5%) and diabetes (9.1%) being the three most frequent conditions. Hospitalisation during the previous 12 months was reported by 15.9% of the participants. The details of the unweighted and weighted sample characteristics for the total sample and individual countries are shown in Table 1.

**Table 1:** Participant sociodemographic and health data.

		Total		Germany		Austria		France	
		Weighted	Unweighted	Weighted	Unweighted	Weighted	Unweighted	Weighted	Unweighted
		(N = 7087)	(N = 7087)	(n = 1016)	(n = 1016)	(n = 1007)	(n = 1007)	(n = 1033)	(n = 1033)
Sex N (%)	Male	3434 (48.4)	3467 (48.9)	494 (48.7)	498 (49.0)	488 (48.5)	492 (48.9)	501 (48.5)	498 (48.2)
	Female	3653 (51.6)	3620 (51.1)	522 (51.3)	518 (51.0)	519 (51.5)	515 (51.1)	532 (51.5)	535 (51.8)
Age (years)	M (SD)	49.6 (16.6)	47.1 (16.1)	50.3 (16.4)	48.2 (16.2)	49.2 (16.8)	46.4 (16.3)	49.7 (16.9)	46.6 (16.1)
	Median (IQR)	50 (36–64)	47 (33–60)	52 (36–64)	49 (34–61)	50 (35–64)	46 (33–59)	50 (35–64)	47 (32–60)
Education N (%)	Compulsory or less	529 (7.5)	510 (7.2)	53 (5.3)	54 (5.3)	68 (6.8)	71 (7.1)	167 (16.2)	168 (16.3)
	Secondary or vocational training	3989 (56.3)	3979 (56.1)	688 (67.7)	691 (68)	750 (74.5)	740 (73.5)	454 (44)	454 (43.9)
	University degree	2569 (36.3)	2598 (36.7)	274 (27)	271 (26.7)	189 (18.8)	196 (19.5)	412 (39.8)	411 (39.8)
Marital status N (%)	Single	1699 (23.9)	1887 (26.6)	245 (24.1)	266 (26.2)	221 (22)	251 (24.9)	315 (30.5)	353 (34.2)
	Married or in a steady relationship	4277 (60.4)	4205 (59.3)	589 (57.9)	584 (57.5)	604 (60)	594 (59)	522 (50.5)	505 (48.9)
	Separated or divorced	797 (11.2)	745 (10.5)	132 (13)	125 (12.3)	137 (13.6)	127 (12.6)	196 (19)	175 (16.9)
	Widowed	315 (4.5)	250 (3.5)	50 (4.9)	41 (4)	45 (4.4)	35 (3.5)	0 (0)	0 (0)
Health condition N (%)	No health condition	4296 (60.6)	3238 (45.7)	602 (59.2)	614 (60.4)	625 (62)	648 (64.3)	544 (52.7)	567 (54.9)
	At least one health condition	2791 (39.4)	2832 (40.0)	414 (40.8)	402 (39.6)	382 (38)	359 (35.7)	489 (47.3)	466 (45.1)
	Asthma, COPD or related	671 (9.5)	674 (9.5)	90 (8.8)	89 (8.8)	93 (9.2)	92 (9.1)	89 (8.6)	89 (8.6)
	Arthritis or rheumatism	778 (11.0)	721 (10.2)	82 (8.1)	80 (7.9)	89 (8.8)	77 (7.6)	178 (17.2)	164 (15.9)
	Cancer (diagnosis in last 3 years)	173 (2.4)	152 (2.2)	18 (1.8)	17 (1.7)	36 (3.6)	29 (2.9)	19 (1.9)	16 (1.5)
	Diabetes	643 (9.1)	575 (8.1)	111 (10.9)	99 (9.7)	86 (8.5)	76 (7.5)	78 (7.5)	66 (6.4)
	Gastrointestinal diseases	434 (6.1)	428 (6.0)	44 (4.3)	44 (4.3)	66 (6.6)	62 (6.2)	43 (4.2)	45 (4.4)
	Heart diseases	506 (7.1)	450 (6.3)	86 (8.5)	80 (7.9)	63 (6.2)	56 (5.6)	81 (7.9)	68 (6.6)
	HIV or AIDS	27 (0.4)	28 (0.4)	4 (0.4)	4 (0.4)	2 (0.2)	2 (0.2)	3 (0.3)	3 (0.3)
	Renal diseases	194 (2.7)	182 (2.6)	25 (2.4)	24 (2.4)	21 (2.1)	21 (2.1)	24 (2.4)	20 (1.9)
	Liver diseases	102 (1.5)	104 (1.5)	14 (1.4)	14 (1.4)	12 (1.2)	12 (1.2)	5 (0.5)	5 (0.5)
	Stroke	104 (1.5)	92 (1.3)	21 (2)	19 (1.9)	17 (1.7)	14 (1.4)	16 (1.5)	14 (1.4)
	Mental health disorder*	435 (7.2)	448 (6.3)	131 (12.9)	138 (13.6)	120 (11.9)	124 (12.3)	47 (4.5)	49 (4.7)
Hospitalization in last 12 months N (%)	Yes	1129 (15.9)	1129 (15.9)	189 (18.6)	187 (18.4)	197 (19.6)	182 (18.1)	169 (16.4)	165 (16.0)
	No	5958 (84.1)	5958 (84.1)	827 (81.4)	829 (81.6)	810 (80.4)	825 (81.9)	864 (83.6)	868 (84.0)
Living situation N (%)	Living alone	1428 (20.1)	1428 (20.1)	320 (31.5)	313 (30.8)	254 (25.2)	250 (24.8)	254 (24.6)	245 (23.7)
	Living with partner	4288 (60.5)	4288 (60.5)	562 (55.3)	555 (54.6)	593 (58.9)	579 (57.5)	627 (60.7)	623 (60.3)
	Living with children	1794 (25.3)	1794 (25.3)	191 (18.8)	204 (20.1)	151 (15)	159 (15.8)	188 (18.2)	207 (20)
	With parents	729 (10.3)	729 (10.3)	52 (5.1)	61 (6)	76 (7.6)	89 (8.8)	54 (5.3)	63 (6.1)
	With siblings	182 (2.6)	182 (2.6)	20 (2)	23 (2.3)	19 (1.9)	23 (2.3)	14 (1.4)	17 (1.6)
	With other family members	212 (3.0)	212 (3.0)	23 (2.3)	24 (2.4)	29 (2.9)	30 (3)	24 (2.3)	22 (2.1)
	Other adult people (non-family)	156 (2.2)	156 (2.2)	24 (2.4)	26 (2.6)	22 (2.2)	26 (2.6)	29 (2.8)	30 (2.9)

		Italy		Poland		Netherlands		Spain	
		Weighted	Unweighted	Weighted	Unweighted	Weighted	Unweighted	Weighted	Unweighted
		(n = 1005)	(n = 1005)	(n = 999)	(n = 999)	(n = 1017)	(n = 1017)	(n = 1010)	(n = 1010)
Sex N (%)	Male	484 (48.1)	492 (49)	475 (47.5)	483 (48.3)	501 (49.3)	504 (49.6)	490 (48.5)	500 (49.5)
	Female	521 (51.9)	513 (51)	524 (52.5)	516 (51.7)	516 (50.7)	513 (50.4)	520 (51.5)	510 (50.5)
Age in years	M (SD)	51.4 (16.3)	48.5 (15.9)	47.5 (16.4)	45.0 (16)	49.5 (17)	47.9 (16.8)	49.7 (15.9)	47.1 (15.5)
	Median (IQR)	52 (39–65)	48 (35–61)	47 (33–61)	44 (31–59)	50 (36–65)	48 (34–62)	49 (37–63)	46 (35–60)
Education N (%)	Compulsory or less	129 (12.8)	112 (11.1)	17 (1.7)	17 (1.7)	19 (1.8)	19 (1.9)	75 (7.4)	69 (6.8)
	Secondary or vocational training	518 (51.6)	521 (51.8)	499 (49.9)	501 (50.2)	597 (58.7)	592 (58.2)	483 (47.8)	480 (47.5)
	University degree	358 (35.6)	372 (37)	483 (48.4)	481 (48.1)	401 (39.5)	406 (39.9)	452 (44.7)	461 (45.6)
Marital status N (%)	Single	208 (20.7)	239 (23.8)	167 (16.7)	183 (18.3)	290 (28.5)	313 (30.8)	253 (25)	282 (27.9)
	Married or in a stead relationship	669 (66.6)	656 (65.3)	689 (68.9)	690 (69.1)	580 (57)	568 (55.9)	625 (61.8)	608 (60.2)
	Separated or divorced	70 (7)	68 (6.8)	76 (7.6)	72 (7.2)	104 (10.2)	99 (9.7)	81 (8)	79 (7.8)
	Widowed	57 (5.7)	42 (4.2)	68 (6.8)	54 (5.4)	44 (4.3)	37 (3.6)	51 (5.1)	41 (4.1)
Health condition N (%)	No health condition	600 (59.7)	614 (61.1)	585 (58.6)	608 (60.9)	685 (67.3)	700 (68.8)	655 (64.8)	663 (65.6)
	At least one health condition	405 (40.3)	391 (38.9)	414 (41.4)	391 (39.1)	332 (32.7)	317 (31.2)	355 (35.2)	347 (34.4)
	Asthma, COPD or related	107 (10.6)	109 (10.8)	81 (8.1)	77 (7.7)	104 (10.2)	105 (10.3)	108 (10.7)	113 (11.2)
	Arthritis or rheumatism	148 (14.8)	138 (13.7)	81 (8.2)	74 (7.4)	114 (11.2)	107 (10.5)	85 (8.5)	81 (8.0)
	Cancer (diagnosis in last 3 years)	31 (3.1)	28 (2.8)	27 (2.7)	24 (2.4)	25 (2.4)	23 (2.3)	18 (1.7)	16 (1.6)
	Diabetes	100 (9.9)	94 (9.4)	94 (9.4)	84 (8.4)	83 (8.2)	75 (7.4)	93 (9.2)	81 (8.0)
	Gastrointestinal diseases	63 (6.3)	64 (6.4)	122 (12.2)	119 (11.9)	41 (4.0)	41 (4.0)	54 (5.4)	53 (5.2)
	Heart diseases	60 (6)	57 (5.7)	119 (11.9)	105 (10.5)	57 (5.6)	50 (4.9)	39 (3.8)	34 (3.4)
	HIV or AIDS	4 (0.4)	4 (0.4)	2 (0.2)	2 (0.2)	6 (0.6)	6 (0.6)	6 (0.6)	7 (0.7)
	Renal diseases	30 (3)	29 (2.9)	53 (5.3)	50 (5)	17 (1.7)	17 (1.7)	24 (2.4)	21 (2.1)
	Liver diseases	23 (2.3)	23 (2.3)	35 (3.5)	35 (3.5)	6 (0.6)	6 (0.6)	8 (0.8)	9 (0.9)
	Stroke	6 (0.6)	5 (0.5)	24 (2.4)	21 (2.1)	11 (1.1)	10 (1.0)	9 (0.9)	9 (0.9)
	Mental health disorder*	55 (5.5)	56 (5.6)	38 (3.8)	35 (3.5)	*	*	44 (4.3)	46 (4.6)
Hospitalization in last 12 months N (%)	Yes	142 (14.1)	147 (14.6)	165 (16.5)	159 (15.9)	182 (17.9)	175 (17.2)	113 (11.2)	114 (11.3)
	No	863 (85.9)	858 (85.4)	834 (83.5)	840 (84.1)	835 (82.1)	842 (82.8)	897 (88.8)	896 (88.7)
Living situation N (%)	Living alone	120 (11.9)	120 (11.9)	150 (15)	136 (13.6)	248 (24.4)	244 (24)	126 (12.5)	120 (11.9)
	Living with partner	651 (64.8)	651 (64.8)	619 (62)	613 (61.4)	624 (61.3)	614 (60.4)	667 (66.1)	653 (64.7)
	Living with children	342 (34)	342 (34)	264 (26.5)	271 (27.1)	208 (20.5)	215 (21.1)	389 (38.5)	396 (39.2)
	With parents	152 (15.1)	152 (15.1)	117 (11.7)	137 (13.7)	72 (7.1)	83 (8.2)	125 (12.3)	144 (14.3)
	With siblings	31 (3.1)	31 (3.1)	24 (2.4)	27 (2.7)	20 (2)	23 (2.3)	33 (3.3)	38 (3.8)
	With other family members	31 (3.1)	31 (3.1)	68 (6.8)	69 (6.9)	21 (2.1)	22 (2.2)	13 (1.3)	14 (1.4)
	Other adult people (non-family)	10 (1)	10 (1)	29 (2.9)	33 (3.3)	12 (1.2)	13 (1.3)	16 (1.5)	18 (1.8)

*In the Netherlands mental health conditions were not assessed.

### Normative data for the K10 and K6 scales by country, sex and age

**Figure 1:**
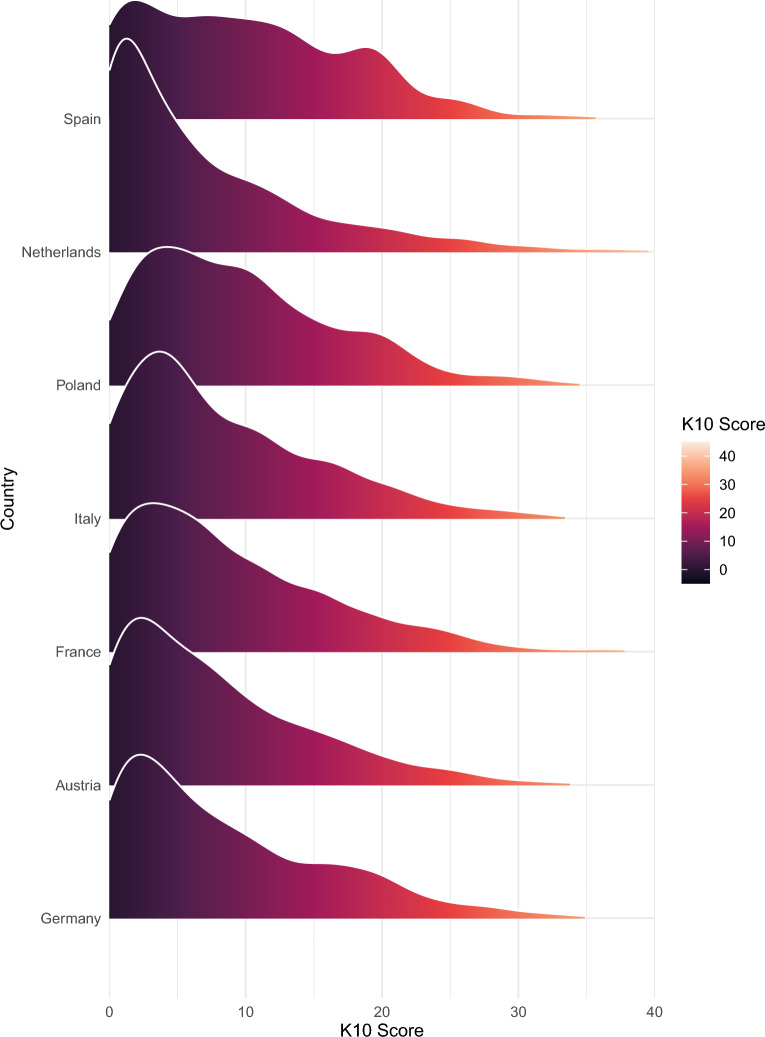
Distribution of Kessler 10 scores by country.

In the weighted total sample, the K10 mean score was 8.5 points (SD = 7.3). The maximum possible score of 40 points was obtained by 0.1% of the participants and the minimum score of 0 points by 9.4% across all countries. The distribution of K10 scores in each country is illustrated in Fig. 1. The mean K10 scores were highest in Spain (9.9 points) and Poland (9.7), followed by France (8.7), Italy (8.4), Germany (8.3), Austria (7.9) and the Netherlands (6.9). Women showed higher K10 mean scores than men across all countries. The largest mean sex differences were found in Germany (+ 2.1 points for women compared to men), Spain (+ 1.9 points for women) and Italy (+ 1.7 points for women).

**Figure 2:**
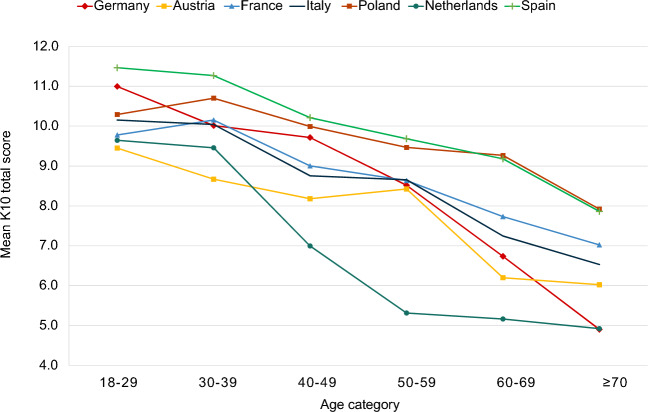
Mean Kessler 10 score by country and age category.

In all seven countries, the two youngest age groups (18–29 and 30–39 years) had the highest K10 mean scores. The largest age-related differences were found in Germany (+ 3.8 points in participants aged 18–29 vs.  > 70 years) and the Netherlands (+ 3.1 points in the 18–29 age group vs. the  > 70 age group). The age trends for the K10 scores are shown in Fig. 2.

The detailed normative data for individual countries and sex and age groups for the K10 scale are shown in Table 2. The normative data for the K6 scale are shown in Supplementary Table 2, while the response frequencies for the individual items of the K10 and K6 scale are reported in Supplementary Table 5.

**Table 2:** K10 normative data (weighted) per country, sex and age group.

Country	Group	K10 score:						
		Mean	SD	10th percentile	25th percentile	50th percentile	75th percentile	90th percentile
Total (N = 7087*)		8.5	7.3	1	3	7	13	19
Germany (n = 1016)		8.3	7.5	1	2	6	13	20
Austria (n = 1007)		7.9	7.0	0	2	6	12	18
France (n = 1033)		8.7	7.3	1	3	7	13	19
Italy (n = 1005)		8.4	7.0	1	3	6	12	18
Poland (n = 999)		9.7	7.0	2	4	8	14	20
Netherlands (n = 1017)		6.9	7.6	0	1	4	10	18
Spain (n = 1010)		9.9	7.5	1	3	9	15	20
Germany (n = 1016)	18–29 years (n = 175)	11.0	7.8	0	5	10	17	21
	30–39 years (n = 165)	10.0	8.7	1	3	8	16	23
	40–49 years (n = 182)	9.7	7.8	1	3	8	16	21
	50–59 years (n = 206	8.5	7.6	1	3	6	13	19
	60–69 years (n = 144)	6.7	5.7	1	2	5	10	15
	≥ 70 years (n = 144)	4.9	5.1	0	1	3	7	12
Austria (n = 1007)	18–29 years (n = 198)	9.5	7.4	1	4	8	14	20
	30–39 years (n = 174)	8.7	8.0	0	3	6	12	20
	40–49 years (n = 193)	8.2	7.7	1	2	6	11	20
	50–59 years (n = 194)	8.4	6.8	1	3	7	13	18
	60–69 years (n = 140)	6.2	5.7	0	2	5	9	15
	≥ 70 years (n = 108)	6.0	5.6	0	1	4	9	15
France (n = 1033)	18–29 years (n = 198)	9.8	7.6	1	4	8	15	22
	30–39 years (n = 176)	10.2	8.0	1	4	8	16	23
	40–49 years (n = 201)	9.0	7.0	2	4	7	13	19
	50–59 years (n = 193)	8.6	7.2	1	3	7	13	19
	60–69 years (n = 161)	7.7	7.1	1	3	6	11	16
	≥ 70 years (n = 104)	7.0	6.4	1	3	4	10	16
Italy (n = 1005)	18–29 years (n = 159)	10.2	7.4	2	5	8	15	21
	30–39 years (n = 159)	10.0	7.7	2	4	8	17	22
	40–49 years (n = 205)	8.8	7.2	2	3	7	13	19
	50–59 years (n = 193)	8.7	7.1	1	3	6	13	18
	60–69 years (n = 155)	7.2	6.6	1	3	5	11	16
	≥ 70 years (n = 134)	6.5	5.6	0	3	5	10	14
Poland (n = 999)	18–29 years (n = 210)	10.3	7.2	2	5	9	14	20
	30–39 years (n = 209)	10.7	7.6	2	5	10	15	21
	40–49 years (n = 165)	10.0	6.9	2	5	9	14	20
	50–59 years (n = 179)	9.5	6.9	2	4	8	14	20
	60–69 years (n = 151)	9.3	6.9	2	3	8	14	18
	≥ 70 years (n = 85)	7.9	6.1	2	4	6	11	19
Netherlands (n = 1017)	18–29 years (n = 190)	9.6	8.4	0	2	8	15	22
	30–39 years (n = 156)	9.5	9.6	0	2	6	15	24
	40–49 years (n = 187)	7.0	7.8	0	1	4	10	19
	50–59 years (n = 198)	5.3	5.8	0	1	3	8	13
	60–69 years (n = 164)	5.2	5.7	0	1	3	8	13
	≥ 70 years (n = 122	4.9	6.3	0	1	3	6	12
Spain (n = 1010)	18–29 years (n = 163)	11.5	7.9	1	4	11	18	22
	30–39 years (n = 184)	11.3	8.1	1	5	10	18	22
	40–49 years (n = 216)	10.2	7.4	2	4	9	14	20
	50–59 years (n = 192)	9.7	6.9	1	3	9	15	20
	60–69 years (n = 143)	9.2	7.2	0	3	8	14	20
	≥ 70 years (n = 112)	7.9	6.9	0	2	7	12	18
Germany (n = 1016)	Men (n = 498)	7.3	7.0	0	2	5	11	18
	Women (n = 518)	9.4	7.8	1	3	7	14	20
Austria (n = 1007)	Men (n = 492)	7.3	6.9	0	2	6	11	17
	Women (n = 515)	8.4	7.1	1	3	7	12	18
France (n = 1033)	Men (n = 498)	8.0	7.3	0	2	6	12	19
	Women (n = 535)	9.3	7.3	1	4	7	14	20
Italy (n = 1005)	Men (n = 492)	7.5	6.6	1	3	5	11	17
	Women (n = 513)	9.2	7.2	1	4	7	13	20
Poland (n = 999)	Men (n = 483)	9.1	7.1	2	3	8	13	19
	Women (n = 516)	10.3	6.9	2	5	9	14	20
Netherlands (n = 1017)	Men (n = 504)	6.3	7.5	0	1	3	10	17
	Women (n = 513)	7.4	7.6	0	2	5	10	18
Spain (n = 1010)	Men (n = 500)	8.9	7.2	0	3	8	14	19
	Women (n = 510)	10.8	7.6	1	5	10	17	20

*The sample sizes as reported in this table refer to the unweighted data. All normative data reported in the table are weighted.

### Regression model for estimating K10 and K6 scores

The univariable linear regression analysis showed that the K10 scores were statistically significantly associated with age (pairwise comparison against the reference ‘18–29 years’ for all age groups [*p* < 0.001] but the ‘30–39 years’ group [*p* = 0.681]), sex (*p* < 0.001), chronic somatic health conditions (*p* < 0.001), chronic mental health conditions (*p* < 0.001), country (pairwise comparisons against the reference ‘Germany’ were statistically significant for Poland [*p* < 0.001], the Netherlands [*p* < 0.001], and Spain [*p* < 0.001]) and educational level (compulsory school education or less differed statistically significantly from secondary or vocational training [*p* = 0.005] and university degree [*p* = 0.002]).

The backward exclusion of predictors in the multivariable linear regression model retained all included variables. For age, all but the ‘30–39 years’ (*p* = 0.152) group differed statistically significantly (*p* ≤ 0.001) from the reference group ‘18–29 years’. Participants with self-reported somatic health conditions (+ 4.02, *p* < 0.001) and women (+ 1.61, *p* < 0.001) showed higher K10 scores. In addition, participants with compulsory education or less had scores that were higher than those from participants with secondary or vocational training (− 1.04, *p* < 0.001) or from those with a university degree (− 1.55, *p* = 0.011). Comparisons of countries against the reference category (Germany) showed statistically significant differences for all countries but Italy (*p* = 0.943) and France (*p* = 0.610). Austria  − 0.62 points (*p* = 0.43) and the Netherlands  − 1.42 points (*p* < 0.001) had lower scores compared to Germany, while Poland  + 0.97 points (*p* = 0.002) and Spain  + 1.68 points (*p* < 0.001) had higher scores. The results are displayed in Table 3; additional results for the K6 scale are given in Supplementary Table 3. Further multivariable regression analyses were done to quantify possible sampling bias (regarding underrepresentation of individuals with mental disorders) by investigating the association between the prevalence of mental disorders and K10 and K6 scores. For the K10 a, for example, 5% higher prevalence of mental disorders in the sample would result in a is 0.43 points higher mean score (please see Supplementary Table 6 for further details).

**Table 3:** Regression model for predicting K10 scores.

Variable	Univariate models			Multivariable model		
	Beta	[95% CI]	*p*-value	Beta	[95% CI]	*p*-value
Constant for multivariable model**				9.77	[8.92; 10.62]	< 0.001
Age (reference: 18–29 years)						
30–39 years	− 0.13	[−0.73. 0.48]	0.681	− 0.42	[−1.00; 0.15]	0.152
40–49 years	− 1.21	[−1.80. − 0.62]	< 0.001	− 1.66	[−2.23; − 1.10]	< 0.001
50–59 years	− 1.85	[−2.44. − 1.26]	< 0.001	− 2.67	[−3.23; − 2.10]	< 0.001
60–69 years	− 2.84	[−3.46. − 2.22]	< 0.001	− 4.19	[−4.79; − 3.60]	< 0.001
70 + years	− 3.80	[−4.40. − 3.21]	< 0.001	− 5.39	[−5.97; − 4.81]	< 0.001
Sex (reference: men)	1.49	[1.14. 1.83]	< 0.001	1.61	[1.29; 1.93]	< 0.001
Somatic health condition	3.22	[2.86. 3.58]	< 0.001	4.02	[3.67; 4.37]	< 0.001
Mental health condition	9.11	[8.44. 9.79]	< 0.001	*	*	*
Country (reference: Germany)						
Austria	− 0.58	[−1.22. 0.06]	0.078	− 0.62	[−1.22; − 0.02]	0.043
France	0.25	[−0.38. 0.89]	0.435	0.16	[−0.44; 0.76]	0.610
Italy	− 0.01	[−0.65. 0.63]	0.971	− 0.02	[−0.62. 0.58]	0.943
Poland	1.18	[0.54. 1.83]	< 0.001	0.97	[0.37; 1.58]	0.002
Netherlands	− 1.64	[−2.28. − 1.00]	< 0.001	− 1.42	[−2.01; − 0.82]	< 0.001
Spain	1.45	[0.81. 2.09]	< 0.001	1.68	[1.08. 2.29]	< 0.001
Education (reference: compulsory or less)						
Secondary or vocational training	− 0.98	[−1.67. − 0.30]	0.005	− 1.04	[−0.41; − 0.07]	0.001
University degree	− 1.09	[−1.79. − 0.38]	0.002	− 1.55	[−0.90; − 0.10]	< 0.001

Dependent variable: K10 score (range 0–40); coding: Sex (men = 0, women = 1); health conditions (no health conditions = 0, at least one health condition = 1); *CI* Confidence interval.

*The variable 'mental health condition' was excluded from the multivariable model to avoid over-adjustment.

**Constants for univariate models are not shown.

### Diagnostic accuracy of the K10 scale for predicting self-reported mental health disorders

We conducted a ROC analysis to investigate the diagnostic accuracy of the K10 scale for predicting self-reported mental health disorders and determined the thresholds. The diagnostic accuracy for this criterion was high across countries with AUC values ranging from 0.77 (Italy) to 0.87 (Germany). The thresholds providing the highest sensitivity and specificity (i.e., maximal Youden J) ranged from 7.5 points (Italy) to 16.5 (Spain). When selecting a cut-off score with at least a sensitivity of 0.80, the cut-off scores ranged from 5.5 (Austria) to 13.5 (France). Additional results are reported in Supplementary Table 1. Details on the analysis for the K6 scale are reported in Supplementary Table 4.

## Discussion

The results of our analysis provide age- and sex-specific general population normative data based on the K10 and K6 scales for seven European countries. Our descriptive analysis found that women and younger participants had higher distress levels than men and older participants across all analysed countries. This association of sex and age with K10 scores was also found using a multivariable regression model adjusted for country, educational level and self-reported somatic health conditions. In this model, the group differences in scores were below 2 points for all analysed variables, except for somatic health conditions and specific age groups. In a separate univariate analysis, we also investigated the differences in K10 scores between participants with and without self-reported mental conditions and found a difference of 9.11 points (about 1.2 SD). This large difference reflects the discriminatory power of the K10 that was also shown in a ROC analysis using self-reported mental conditions as criterion. In this analysis, the diagnostic accuracy in terms of AUC and the optimal cut-off scores varied substantially across countries similarly to the prevalence of these self-reported conditions.

We sampled and weighted the collected sample to match the sex and age distributions in the respective countries. The other sample characteristics were largely aligned with the available data^31,32^, with education level being the most notable exception. The comparison of the distribution of educational levels in our samples against the general population was challenging because of the limited availability of detailed international data and variation within educational systems. After comparing our data with OECD data, however, we identified an over-representation of higher educated individuals in our sample^33^. While using an online panel data company to collect data is a common technique for collecting normative data, sampling biases regarding educational levels in this recruitment strategy have been reported previously^34^. However, this bias may be of limited importance because of the rather small association of K10 scores with education level in our multivariable analysis results. In our samples, the lack of data on self-reported mental health disorders that can be compared against national data is a more important limitation because the definitions of these disorders differ to some degree across studies, which compromises our conclusions about their possible differences. In addition, individuals with mental health disorders may be less likely to participate in online surveys (please note that this might also be a source of bias for community health studies relying on a similar assessment methodologies). Therefore, we provided multivariable regression models that allow to estimate normative scores as a function of prevalence of mental health disorders.

Following health conditions, age was found to have the strongest association with K10 scores, which is consistent with studies that also reported lower distress levels in older individuals^35–37^. Sex differences regarding psychological distress have also been described consistently in the literature in relation to biological determinants^38^ and social factors but have also been reported to be context-specific^26^. It is noteworthy that the impact of sex on K10 scores in our large general population dataset was small in relation to the participants’ other characteristics. These results may partially reflect the sex invariance in the construct validity of the scale^39,40^, which suggests that the observed differences in K10 scores may reflect the true differences in psychological distress rather than being a result of the variation in measurement characteristics or response styles that may inflate the actual differences between women and men.

The variation of K10 mean scores across countries was substantial, with a similar magnitude in the difference between the Netherlands and Spain to the difference between individuals with and without somatic health conditions. When we compared our results against normative data from the literature, the scores in European countries were higher than in the Australian general population^41^ with its reported K10 mean score of 4.5 points (on a 0–40 metric), while age and sex differences were of the same magnitude. Even lower mean scores were observed in a Swiss community study (random sampling of adults aged 19–45 years) that found a mean 2.5 score for the K10 scale (on a 0–40 metric). However, the comparison of K10 scores across countries is compromised by the variation in sampling methodologies. Therefore, the uniform data collection approach in our study is a major strength because it improves the comparability of mean scores across various countries, which in turn supports the importance of collecting country-specific normative data.

Cross-cultural variation has been shown for the K10 scale not only in normative data but also in its measurement characteristics and screening properties. The K10 scale was originally developed in the English language for use in the US and Canada^10^, followed by large population studies in Australia^13^. While the development of the scale relied on sophisticated psychometric methods, it did not seem to focus on cross-cultural applicability. In their extensive review of the evidence for cultural equivalence and measurement characteristics, Stolk et al.^15^ highlighted the substantial variation in the factor structure or acceptability of item wording (in particular in non-Western and non-white populations) for example but did not indicate substantial differential item function for the K10 score. While this comprehensive review highlights a number of issues with cross-cultural applications of the K10 scale, it also reflects its very widespread use within a short period after its publication.

Our data were collected before the onset of the COVID-19 pandemic. A potential concern would be how the pandemic has shaped psychological distress in the general population. While some studies suggest that there were immediate increases in general population psychological distress during the first months of the pandemic^42,43^, a meta-analysis of longitudinal studies found only small and heterogeneous effects^44^. Moreover, longitudinal survey data indicates that no enduring or sustained effect on common mental health problems or psychological distress was present after the first two lockdowns and psychological returned to baseline (ie, pre-pandemic) levels^42^.

Our study is the first to collect multinational normative data for the K10 and K6 scales from European countries using a consistent sampling approach. These normative data facilitate more meaningful interpretations of patient- or group-level K10 and K6 data in the European setting. In addition, these data can inform health-care professionals, researchers and policymakers about the levels of general distress in groups of individuals with specific characteristics. Furthermore, the data facilitate the interpretation of scores from clinical populations or in clinical studies and may also be used to estimate the pre-disease distress levels in a mental health context. By relying on the uniform data collection and sampling methods in all countries, our data can also be used in country comparisons.

### Supplementary Information

Supplementary Tables.

## Data Availability

Data will not be shared via a public data repository. Data may be available upon reasonable request form the corresponding author.
