# Supplementary material for: General population normative data from seven European countries for the K10 and K6 scales for psychological distress
Source: Sci Rep. 2023 Oct 26;13:18389. doi: 10.1038/s41598-023-45124-0 (PMC10603032; doi:10.1038/s41598-023-45124-0)
Supplement: Supplementary file 1 — Supplementary Tables. [file 41598_2023_45124_MOESM1_ESM.docx]

# Supplementary materials

Supplementary Table 1. ROC analysis and K10 cut-off scores for self-reported mental health disorders

| Country | AUC | [95% CI] | p-value | Cut-off with maximum Youden J* | Sensitivity/Specificity | Cut-off with a sensitivity >0.80 and highest possible Youden J* | Sensitivity/Specificity |
| --- | --- | --- | --- | --- | --- | --- | --- |
| Germany | 0.872 | [0.844; 0.900] | <0.001 | 10.5 | 0.87/0.75 | 10.5 | 0.87/0.75 |
| Austria | 0.787 | [0.743; 0.830] | <0.001 | 11.5 | 0.66/0.79 | 5.5 | 0.82/0.49 |
| France | 0.856 | [0.801; 0.911] | <0.001 | 13.5 | 0.82/0.79 | 13.5 | 0.82/0.79 |
| Italy | 0.768 | [0.712; 0.823] | <0.001 | 7.5 | 0.88/0.57 | 8.5 | 0.80/0.61 |
| Poland | 0.774 | [0.697; 0.851] | <0.001 | 11.5 | 0.77/0.67 | 8.5 | 0.80/0.50 |
| Spain | 0.807 | [0.741; 0.874] | <0.001 | 16.5 | 0.70/0.80 | 11.5 | 0.80/0.62 |

Note: AUC = Area under Curve. In the Netherlands, mental health conditions were not assessed.

* Youden J = sum of sensitivity and specificity minus 1

Supplementary Table 2: K6 normative data (weighted) per country, sex and age group

|  |  | K6 score: | | | | | | |
| --- | --- | --- | --- | --- | --- | --- | --- | --- |
| Country | Group | Mean | SD | 10^th^ percentile | 25^th^ percentile | 50^th^ percentile | 75^th^ percentile | 90^th^ percentile |
| Total (n=7087) |  | 5.2 | 4.6 | 0 | 2 | 4 | 8 | 12 |
| Germany (n=1016) |  | 4.9 | 4.7 | 0 | 1 | 3 | 7 | 12 |
| Austria (n=1007) |  | 4.6 | 4.4 | 0 | 1 | 3 | 7 | 11 |
| France (n=1033) |  | 5.1 | 4.6 | 0 | 2 | 4 | 8 | 12 |
| Italy (n=1005) |  | 5.3 | 4.3 | 1 | 2 | 4 | 8 | 12 |
| Poland (n=999) |  | 6.2 | 4.5 | 1 | 3 | 5 | 9 | 12 |
| Netherlands (n=1017) |  | 4.1 | 4.8 | 0 | 0 | 2 | 6 | 11 |
| Spain (n=1010) |  | 6.1 | 4.7 | 0 | 2 | 6 | 9 | 12 |
| Germany (n=1016) | 18-29 years | 6.6 | 4.9 | 0 | 2 | 6 | 10 | 13 |
|  | 30-39 years | 6.0 | 5.5 | 0 | 2 | 4 | 10 | 14 |
|  | 40-49 years | 5.7 | 5.1 | 0 | 2 | 4 | 10 | 13 |
|  | 50-59 years | 4.9 | 4.8 | 0 | 1 | 3 | 7 | 12 |
|  | 60-69 years | 3.8 | 3.5 | 0 | 1 | 3 | 6 | 9 |
|  | ≥70 years | 2.8 | 3.2 | 0 | 0 | 2 | 4 | 7 |
| Austria (n=1007) | 18-29 years | 5.7 | 4.7 | 0 | 2 | 5 | 9 | 12 |
|  | 30-39 years | 5.1 | 5.1 | 0 | 1 | 3 | 7 | 12 |
|  | 40-49 years | 4.7 | 4.9 | 0 | 1 | 3 | 7 | 12 |
|  | 50-59 years | 5.0 | 4.3 | 0 | 2 | 4 | 8 | 11 |
|  | 60-69 years | 3.7 | 3.7 | 0 | 1 | 3 | 5 | 9 |
|  | ≥70 years | 3.5 | 3.4 | 0 | 1 | 2 | 5 | 9 |
| France (n=1033) | 18-29 years | 5.8 | 4.7 | 0 | 2 | 5 | 9 | 13 |
|  | 30-39 years | 6.0 | 5.1 | 0 | 2 | 4 | 10 | 13 |
|  | 40-49 years | 5.2 | 4.4 | 0 | 2 | 4 | 8 | 12 |
|  | 50-59 years | 5.1 | 4.5 | 0 | 1 | 4 | 8 | 12 |
|  | 60-69 years | 4.6 | 4.5 | 1 | 1 | 3 | 6 | 10 |
|  | ≥70 years | 4.2 | 4.1 | 0 | 1 | 3 | 6 | 10 |
| Italy (n=1005) | 18-29 years | 6.4 | 4.5 | 1 | 3 | 5 | 8 | 14 |
|  | 30-39 years | 6.3 | 4.8 | 1 | 2 | 5 | 10 | 13 |
|  | 40-49 years | 5.5 | 4.4 | 1 | 2 | 4 | 8 | 12 |
|  | 50-59 years | 5.6 | 4.4 | 1 | 2 | 4 | 9 | 12 |
|  | 60-69 years | 4.6 | 4.1 | 0 | 2 | 3 | 7 | 10 |
|  | ≥70 years | 4.3 | 3.6 | 0 | 2 | 4 | 6 | 9 |
| Poland (n=999) | 18-29 years | 6.6 | 4.6 | 1 | 3 | 6 | 9 | 12 |
|  | 30-39 years | 6.8 | 4.8 | 1 | 3 | 6 | 10 | 13 |
|  | 40-49 years | 6.2 | 4.3 | 1 | 3 | 6 | 9 | 12 |
|  | 50-59 years | 6.0 | 4.4 | 1 | 2 | 5 | 9 | 13 |
|  | 60-69 years | 5.9 | 4.5 | 1 | 2 | 5 | 9 | 12 |
|  | ≥70 years | 5.2 | 3.9 | 1 | 2 | 4 | 7 | 12 |
| Netherlands (n=1017) | 18-29 years | 5.9 | 5.3 | 0 | 1 | 5 | 9 | 14 |
|  | 30-39 years | 5.7 | 6.0 | 0 | 1 | 3 | 8 | 15 |
|  | 40-49 years | 4.2 | 4.9 | 0 | 0 | 3 | 6 | 12 |
|  | 50-59 years | 3.1 | 3.6 | 0 | 0 | 2 | 5 | 8 |
|  | 60-69 years | 3.1 | 3.6 | 0 | 0 | 2 | 5 | 8 |
|  | ≥70 years | 2.8 | 3.9 | 0 | 0 | 2 | 4 | 8 |
| Spain (n=1010) | 18-29 years | 7.1 | 5.1 | 0 | 3 | 7 | 11 | 14 |
|  | 30-39 years | 6.9 | 5.0 | 0 | 3 | 7 | 10 | 13 |
|  | 40-49 years | 6.3 | 4.6 | 0 | 2 | 6 | 9 | 13 |
|  | 50-59 years | 6.0 | 4.4 | 0 | 2 | 6 | 9 | 12 |
|  | 60-69 years | 5.7 | 4.6 | 0 | 1 | 5 | 9 | 12 |
|  | ≥70 years | 4.9 | 4.4 | 0 | 1 | 4 | 8 | 11 |
| Germany (n=1016) | Male | 4.2 | 4.4 | 0 | 1 | 3 | 7 | 11 |
|  | Female | 5.5 | 4.9 | 0 | 2 | 4 | 8 | 12 |
| Austria (n=1007) | Male | 4.4 | 4.3 | 0 | 1 | 3 | 7 | 11 |
|  | Female | 4.9 | 4.5 | 0 | 1 | 4 | 7 | 11 |
| France (n=1033) | Male | 4.8 | 4.5 | 0 | 1 | 4 | 7 | 12 |
|  | Female | 5.4 | 4.6 | 1 | 2 | 4 | 8 | 12 |
| Italy (n=1005) | Male | 4.8 | 4.1 | 1 | 2 | 4 | 7 | 11 |
|  | Female | 5.9 | 4.5 | 1 | 3 | 5 | 8 | 13 |
| Poland (n=999) | Male | 5.8 | 4.5 | 1 | 2 | 5 | 8 | 12 |
|  | Female | 6.5 | 4.4 | 1 | 3 | 6 | 9 | 13 |
| Netherlands (n=1017) | Male | 3.7 | 4.7 | 0 | 0 | 2 | 6 | 11 |
|  | Female | 4.4 | 4.8 | 0 | 1 | 3 | 6 | 12 |
| Spain (n=1010) | Male | 5.5 | 4.6 | 0 | 2 | 5 | 9 | 12 |
|  | Female | 6.7 | 4.8 | 0 | 3 | 6 | 10 | 13 |

Supplementary Table 3: Regression model for predicting K6 scores

|  | Univariate models | | | Multivariable model | | |
| --- | --- | --- | --- | --- | --- | --- |
| Variable | Beta | [95% CI] | p-value | Beta | 95% CI | p-value |
| (Constant for multivariable model**) |  |  |  | 5.82 | [5.28; 6.35] | <0.001 |
| Age (reference 18-29years) |  |  |  |  |  |  |
| 30-39 years | -0.15 | [-0,53, 0,24] | 0,455 | -0.34 | [-0.71; 0.02] | 0.067 |
| 40-49 years | -0.84 | [-1,21, -0,47] | <0.001 | -1.13 | [-1.49; -0.77] | <0.001 |
| 50-59 years | -1.19 | [-1,56, -0,81] | <0.001 | -1.68 | [-2.04; -1.32] | <0.001 |
| 60-69 years | -1.77 | [-2,16, -1,38] | <0.001 | -2.59 | [-2.97; -2.21] | <0.001 |
| 70+ years | -2.34 | [-2,71, -1,96] | <0.001 | -3.30 | [-3.66; -2.93] | <0.001 |
| Sex (reference: men) | 0.89 | [0.67, 1.10] | <0.001 | 0.96 | [0.76; 1.16] | <0.001 |
| Somatic health condition | 1.95 | [1.73, 2.18] | <0.001 | 2.43 | [2.21; 2.65] | <0.001 |
| Mental health condition | 5.62 | [5.20, 6.05] | <0.001 | * | * | * |
| Country (reference: Germany) |  | | | | | |
| Austria | -0.29 | [-0.70, 0.11] | 0.152 | -0.31 | [-0.69; 0.07] | 0.106 |
| France | 0.17 | [-0.23, 0.57] | 0.407 | 0.12 | [-0.26; 0.50] | 0.525 |
| Italy | 0.415 | [0.01, 0.82] | 0.044 | 0.43 | [0.05; 0.81] | 0.028 |
| Poland | 1.19 | [0.79, 1.60] | <0.001 | 1.07 | [0.69; 1.45] | <0.001 |
| Netherlands | -0.88 | [-1.28, -0.48] | <0.001 | -0.74 | [-1.12; -0.36] | <0.001 |
| Spain | 1.18 | [0.78, 1.59] | <0.001 | 1.34 | [0.96; 1.72] | <0.001 |
| Education (reference: compulsory or less) |  | | | | | |
| Secondary or vocational training | -0.59 | [-1.03, -0.16] | 0.007 | -0.65 | [-1.05; -0.25] | 0.002 |
| University degree | -0.60 | [-1.05, -0.16] | 0.002 | -0.94 | [-1.36; -0.51] | <0.001 |

Note. Dependent variable: K6 score (range 0–24); coding: Sex (men=0, women=1); health conditions (no health conditions=0, at least one health condition=1); CI: Confidence interval;

* The variable 'mental health condition' was excluded from the multivariable model to avoid over-adjustment

** Constants for univariate models are not shown.

Supplementary Table 4. ROC analysis and K6 cut-off scores for self-reported mental health disorders

| Country | AUC | [95% CI] | p-value | Cut-off with maximum Youden J* | Sensitivity/Specificity | Cut-off with a sensitivity >0.80 and highest possible Youden J* | Sensitivity/Specificity |
| --- | --- | --- | --- | --- | --- | --- | --- |
| Germany | 0.876 | [0.848; 0.905] | <0.001 | 6.5 | 0.84/0.77 | 6.5 | 0.84/0.77 |
| Austria | 0.779 | [0.734; 0.824] | <0.001 | 7.5 | 0.65/0.82 | 2.5 | 0.86/0.45 |
| France | 0.854 | [0.799; 0.910] | <0.001 | 7.5 | 0.84/0.76 | 7.5 | 0.84/0.76 |
| Italy | 0.752 | [0.693; 0.811] | <0.001 | 7.5 | 0.88/0.53 | 7.5 | 0.88/0.53 |
| Poland | 0.750 | [0.669; 0.831] | <0.001 | 7.5 | 0.69/0.68 | 5.5 | 0.83/0.51 |
| Spain | 0.801 | [0.735; 0.866] | <0.001 | 8.5 | 0.76/0.71 | 6.5 | 0.83/0.58 |

Note: AUC = Area under Curve. In the Netherlands, mental health conditions were not assessed.

* Youden J = sum of sensitivity and specificity minus 1

Supplementary Table 5: Relative frequencies of the responses to individual K10 items (weighted data)

|  |  | Total | Germany | Austria | France | Italy | Poland | Netherlands | Spain |
| --- | --- | --- | --- | --- | --- | --- | --- | --- | --- |
| K10 item: How often did you feel… | Response option | (N=7087) | (n=1016) | (n=1007) | (n=1033) | (n=1005) | (n=999) | (n=1017) | (n=1010) |
|  |  | % | % | % | % | % | % | % | % |
| … tired out for no good reason? | None of the time | 24.2 | 20.1 | 24.8 | 23.7 | 27.4 | 16.3 | 34.1 | 22.9 |
|  | A little of the time | 35.8 | 33.8 | 34.6 | 34.2 | 43.0 | 40.3 | 35.5 | 29.4 |
|  | Some of the time | 29.7 | 32.5 | 28.9 | 30.2 | 24.1 | 32.3 | 19.0 | 41.2 |
|  | Most of the time | 8.4 | 11.3 | 9.8 | 9.4 | 4.2 | 9.8 | 8.4 | 5.8 |
|  | All of the time | 1.9 | 2.4 | 1.9 | 2.5 | 1.4 | 1.2 | 3.0 | 0.6 |
| … nervous? | None of the time | 23.4 | 27.8 | 24.5 | 19.8 | 15.4 | 9.1 | 42.6 | 24.4 |
|  | A little of the time | 38.3 | 37.3 | 38.9 | 33.3 | 45.5 | 45.7 | 38.2 | 29.3 |
|  | Some of the time | 29.7 | 28.1 | 29.5 | 34.7 | 31.1 | 32.4 | 13.7 | 38.7 |
|  | Most of the time | 7.0 | 5.6 | 6.0 | 10.0 | 5.8 | 11.0 | 4.4 | 6.4 |
|  | All of the time | 1.6 | 1.3 | 1.2 | 2.2 | 2.2 | 1.7 | 1.2 | 1.2 |
| … so nervous that nothing could calm you down? | None of the time | 61.7 | 69.7 | 73.6 | 56.3 | 57.8 | 48.5 | 66.8 | 59.2 |
|  | A little of the time | 23.3 | 18.6 | 16.8 | 26.3 | 26.9 | 32.9 | 20.0 | 22.1 |
|  | Some of the time | 11.5 | 8.8 | 7.3 | 12.8 | 12.3 | 15.2 | 9.0 | 15.0 |
|  | Most of the time | 2.7 | 2.7 | 1.8 | 3.9 | 2.2 | 3.0 | 3.1 | 2.5 |
|  | All of the time | 0.7 | 0.3 | 0.5 | 0.8 | 0.9 | 0.4 | 1.1 | 1.3 |
| … hopeless? | None of the time | 57.7 | 56.8 | 60.6 | 60.7 | 65.2 | 39.6 | 65.7 | 55.0 |
|  | A little of the time | 22.8 | 19.6 | 21.7 | 21.7 | 22.4 | 33.2 | 20.1 | 21.0 |
|  | Some of the time | 14.2 | 16.6 | 12.9 | 12.8 | 10.1 | 20.0 | 8.9 | 18.3 |
|  | Most of the time | 4.0 | 5.5 | 3.8 | 3.5 | 2.0 | 5.1 | 3.7 | 4.3 |
|  | All of the time | 1.3 | 1.5 | 1.0 | 1.4 | 0.3 | 2.1 | 1.6 | 1.5 |
| … restless or fidgety? | None of the time | 37.4 | 48.0 | 42.7 | 51.5 | 23.4 | 19.3 | 47.5 | 28.6 |
|  | A little of the time | 35.7 | 28.4 | 32.9 | 28.6 | 48.1 | 47.0 | 35.5 | 29.8 |
|  | Some of the time | 21.0 | 17.7 | 19.6 | 15.8 | 22.8 | 25.5 | 11.5 | 34.2 |
|  | Most of the time | 4.9 | 5.1 | 3.9 | 3.2 | 4.5 | 7.2 | 3.9 | 6.5 |
|  | All of the time | 1.1 | 0.7 | 1.0 | 1.0 | 1.3 | 1.0 | 1.6 | 0.9 |
| … so restless that you could not sit still? | None of the time | 62.8 | 66.1 | 70.0 | 66.6 | 60.3 | 49.4 | 71.4 | 55.1 |
|  | A little of the time | 21.2 | 19.8 | 18.4 | 19.1 | 24.7 | 33.1 | 17.7 | 15.5 |
|  | Some of the time | 11.2 | 11.1 | 8.6 | 10.5 | 11.6 | 13.6 | 7.2 | 15.9 |
|  | Most of the time | 4.0 | 2.4 | 2.7 | 2.9 | 2.9 | 3.2 | 2.9 | 10.8 |
|  | All of the time | 0.9 | 0.7 | 0.3 | 0.8 | 0.5 | 0.6 | 0.9 | 2.6 |
| … depressed? | None of the time | 45.3 | 37.2 | 33.0 | 36.1 | 49.8 | 57.3 | 57.2 | 46.6 |
|  | A little of the time | 28.5 | 28.9 | 34.9 | 32.0 | 30.0 | 24.6 | 25.2 | 23.7 |
|  | Some of the time | 19.0 | 24.7 | 23.1 | 23.3 | 15.6 | 12.3 | 9.8 | 24.2 |
|  | Most of the time | 5.7 | 7.3 | 7.6 | 6.8 | 3.9 | 4.5 | 5.6 | 4.1 |
|  | All of the time | 1.5 | 1.9 | 1.3 | 1.7 | 0.8 | 1.3 | 2.2 | 1.4 |
| … so depressed that nothing could cheer you up? | None of the time | 49.3 | 65.5 | 67.7 | 62.1 | 55.6 | 44.7 | 69.1 | 36.1 |
|  | A little of the time | 26.5 | 18.1 | 17.9 | 19.7 | 27.8 | 34.0 | 16.4 | 25.5 |
|  | Some of the time | 15.6 | 11.6 | 11.0 | 13.6 | 11.3 | 16.2 | 9.7 | 24.9 |
|  | Most of the time | 6.5 | 3.9 | 2.8 | 4.0 | 4.2 | 4.2 | 3.2 | 10.0 |
|  | All of the time | 2.1 | 0.9 | 0.6 | 0.8 | 1.1 | 0.8 | 1.5 | 3.5 |
| … that everything was an effort? | None of the time | 54.2 | 42.6 | 48.7 | 46.4 | 38.5 | 42.8 | 49.1 | 55.1 |
|  | A little of the time | 24.6 | 29.7 | 27.8 | 27.3 | 35.4 | 34.0 | 29.2 | 14.8 |
|  | Some of the time | 14.2 | 19.2 | 15.4 | 16.3 | 17.8 | 14.8 | 12.1 | 14.9 |
|  | Most of the time | 5.6 | 7.2 | 6.4 | 8.0 | 6.6 | 7.1 | 7.2 | 9.8 |
|  | All of the time | 1.5 | 1.3 | 1.7 | 2.0 | 1.7 | 1.2 | 2.5 | 5.5 |
| … worthless? | None of the time | 60.5 | 65.8 | 66.5 | 56.0 | 55.1 | 53.4 | 67.4 | 59.4 |
|  | A little of the time | 19.9 | 15.1 | 16.8 | 20.1 | 24.9 | 26.8 | 16.6 | 19.4 |
|  | Some of the time | 13.0 | 12.6 | 11.2 | 15.0 | 13.2 | 13.7 | 9.3 | 15.5 |
|  | Most of the time | 4.6 | 4.1 | 4.0 | 6.5 | 5.0 | 4.2 | 4.7 | 3.5 |
|  | All of the time | 2.0 | 2.4 | 1.5 | 2.4 | 1.8 | 1.9 | 2.0 | 2.2 |

Supplementary Table 6: Multivariate regression model for predicting K10 and K6 scores including mental health conditions

|  | K10 | | | K6 | | |
| --- | --- | --- | --- | --- | --- | --- |
| Variable | Beta | [95% CI] | p-value | Beta | [95% CI] | p-value |
| Constant | 8.26 | 7.43 - 9.09 | >0.001 | 4.87 | 4.34 - 5.39 | >0.001 |
| Age (reference: 18-29years) |  |  |  |  |  |  |
| 30-39 years | -0.39 | -0.98 - 0.20 | 0.193 | -0.32 | -0.69 - 0.05 | 0.094 |
| 40-49 years | -1.67 | -2.24 - -1.09 | >0.001 | -1.15 | -1.51 - -0.78 | >0.001 |
| 50-59 years | -2.32 | -2.89 - -1.74 | >0.001 | -1.46 | -1.82 - -1.09 | >0.001 |
| 60-69 years | -3.69 | -4.31 - -3.08 | >0.001 | -2.29 | -2.68 - -1.90 | >0.001 |
| 70+ years | -4.76 | -5.35 - -4.17 | >0.001 | -2.88 | -3.26 - -2.51 | >0.001 |
| Sex (reference: men) | 1.53 | 1.20 - 1.86 | >0.001 | 0.91 | 0.70 - 1.11 | >0.001 |
| Somatic health condition | 3.58 | 3.23 - 3.94 | >0.001 | 2.16 | 1.94 - 2.38 | >0.001 |
| Mental health condition | 8.63 | 7.99 - 9.27 | >0.001 | 5.42 | 5.01 - 5.83 | >0.001 |
| Country (reference: Germany)* |  | | | | | |
| Austria | -0.50 | -1.06 - 0.07 | 0.083 | -0.24 | -0.59 - 0.12 | 0.195 |
| France | 0.90 | 0.33 - 1.47 | 0.002 | 0.59 | 0.23 - 0.95 | 0.001 |
| Italy | 0.63 | 0.07 - 1.20 | 0.029 | 0.84 | 0.48 - 1.20 | >0.001 |
| Poland | 1.77 | 1.20 - 2.34 | >0.001 | 1.57 | 1.21 - 1.93 | >0.001 |
| Spain | 2.40 | 1.83 - 2.97 | >0.001 | 1.79 | 1.43 - 2.15 | >0.001 |
| Education (reference: compulsory or less) |  | | | | | |
| Secondary or vocational training | -0.77 | -1.38 - -0.16 | 0.014 | -0.48 | -0.86 - -0.09 | 0.016 |
| University degree | -1.07 | -1.70 - -0.43 | 0.001 | -0.64 | -1.04 - -0.24 | 0.002 |
| Note: Dependent variable: K10 score (range 0–40) and K6 score (range 0-24); coding: Sex (men=0, women=1); health conditions (no health conditions=0, at least one health condition=1); CI: confidence interval.  * The Netherlands were not included in this analysis as no self-reported mental health conditions were assessed for the Dutch population. | | | | | | |
